# Providing Psychological and Emotional Support After Perinatal Loss: Protocol for a Virtual Reality-Based Intervention

**DOI:** 10.3389/fpsyg.2020.01262

**Published:** 2020-06-10

**Authors:** Giulia Corno, Stéphane Bouchard, Rosa M. Baños, Marie-Christine Rivard, Chantal Verdon, Francine de Montigny

**Affiliations:** ^1^Département de Psychoéducation et de Psychologie, Université du Québec en Outaouais, Gatineau, QC, Canada; ^2^Centre de Santé et de Services Sociaux de l’Outaouais, Gatineau, QC, Canada; ^3^Departamento Personalidad, Evaluación y Tratamientos Psicológicos, Universitat de València, Valencia, Spain; ^4^CIBER Fisiopatología Obesidad y Nutrición (CIBEROBN), Instituto Carlos III, Madrid, Spain; ^5^Département des Sciences Infirmières, Université du Québec en Outaouais, Gatineau, QC, Canada

**Keywords:** perinatal loss and grief, intervention, virtual reality, women mental health, psychological intervention

## Abstract

The loss of an infant during the perinatal period has been recognized as a complex and potentially traumatic life event and can have a significant impact on women’s mental health. However, often times, psychological aftercare is typically not offered, and manualized interventions are rarely used in clinical care practice and have seldom been evaluated. In recent years, a growing number of studies have demonstrated the efficacy of virtual reality (VR) interventions to facilitate the expression and coping with emotions linked to a traumatic event. The objective of the proposed paper is to present the protocol of a randomized control trial aimed to assess a novel VR-based intervention for mothers who experienced a perinatal loss. We hypothesize that the VR-based intervention group will show significantly reduced symptoms related to grief, postnatal depression and general psychopathology after treatment relative to a treatment-as-usual (TAU) group. Participants would be randomly assigned to the TAU + VR or to the VR + TAU condition. The TAU condition as well as the VR-based intervention will last 3 weeks, after which women will complete a post-assessment. The proposed VR-based intervention will consist in three weekly sessions focused, respectively on: (1) collect information about the loss and psychoeducation about perinatal grief, and introduction to the virtual environment; (2) through the use of the virtual environment, women will be assisted in the elaboration and acceptation of loss; (3) recreate, using the specific features of the virtual environment a positive metaphor representing woman’s future life. VR has proved to be a valid intervention tool in clinical psychology, and in the last years VR technologies have become more affordable to be used in clinical practice. With the present study we propose to answer to the unquestionable need for interventions addressed to ameliorate the emotional effects in women who experienced perinatal loss, by exploiting also the therapeutic opportunities offered by a new technology as VR.

## Introduction

The perinatal period is a complex time in women’s lives. It is a transitional time which requires important changes and challenges as searching for a new identity as an individual, a partner in a couple, and a member of a society (Bibring, 1959; Barclay et al., 1997). The complexity of this period can dramatically increase if women experience perinatal loss. The loss of an infant during the perinatal period has been recognized as complex emotional experience and a potentially traumatic life event for women who experienced it, no matter how and at what gestational stage it occurred (e.g., Berman, 2001; Klier et al., 2002; Bennett et al., 2012). Indeed, although mothers have few or no direct life experiences with their infant, grief in a context of the loss of a child in the perinatal period does not vary significantly in terms of intensity from other type of loss (Kersting and Wagner, 2012). In this paper we use the term perinatal loss to refer to the experiences of women who lost a child during the perinatal period (i.e., loss due to miscarriage, stillbirth, termination due to medical indications, and neonatal death). Miscarriage, stillbirth, and neonatal death continue to be significant topics of concern in the twenty-first century. In Québec (a province of Canada) it is estimated that each year 20,000 pregnancies do not terminate in a live birth (Agence de la santé publique du Canada, 2008).

Research and clinical reports have shown that the loss of a child can cause a multitude of emotions and psychological distress in many women (de Montigny et al., 2017b). Grief is a natural, non-pathological phenomenon and a deeply personal process (Kersting and Wagner, 2012). Reactions to the loss of a significant one can involve transient impairment of daily functioning, elusion of social activities, presence of recurrent and intrusive thoughts, feelings of deep longing and incapability of feeling sensations and emotions (Kersting and Wagner, 2012). The severity of psychological distress generally tends to wane over the first year after the loss (e.g., Bennett et al., 2012). Nonetheless about 20% of women continue to suffer from clinically significant symptoms 1 year after their loss (Boyle et al., 1996; Leon, 2001). Perinatal losses have been shown to have detrimental effects on both mothers’ and fathers’ psychological well-being, on the other members of their families, and are also associated with post-traumatic stress, depression, anxiety, and sleep disturbances (Boyle et al., 1996; Hughes and Riches, 2003). Hughes et al. (2002) reported that approximately 20% of women who experienced a perinatal loss can manifest depression or posttraumatic stress symptomatology. Furthermore, losing a child during the perinatal period has been identified as a complex and potential threatful life experience, which can, in some cases, further develop in complicated grief reactions, characterized by more disruptive, pervasive, or stable symptoms than in a normal grief response. Persistent complex bereavement disorder, also known as complicated grief disorder, has been included in the Diagnostic and Statistical Manual of Mental Disorders, 5th Edition (DSM-5; American Psychiatric Association, 2013).

Despite the increasing recognition that perinatal loss can lead to significant consequences on women’s mental health, often times, few of the women affected receive emotional and psychological support (de Montigny et al., 2017b). The necessity of psychological interventions supporting women after a pregnancy loss is unquestioned in the scientific literature. Nevertheless, psychological aftercare is typically not offered, and manualized interventions are hardly applied in clinical care practice (e.g., Kersting and Wagner, 2012; de Montigny et al., 2017b). The lack of manualized interventions mirrors the corresponding lack in the scientific literature. Indeed, in an attempt to systematically review the scientific literature for psychological and support interventions to reduce levels of stress, anxiety or depression on women who experienced a miscarriage, Campillo et al. (2017) did not find any randomized controlled trials.

In recent years, new technologies have been increasingly used for the assessment, treatment and better understanding of mental health related problems. For instance, Internet- and computer-based interventions are increasingly popular, ranging from pure psychoeducation websites to online self-help groups, self-help interventions, counseling, and even psychotherapy for all sorts of mental health problems (e.g., Andersson and Titov, 2014; Ebert et al., 2018; Moshe et al., 2020), including for parents who experiences perinatal loss (e.g., Kersting et al., 2011a, b). Virtual reality (VR) is another technology that saw an exponential increasing in its popularity in clinical and health psychology. VR embodied the unique opportunity to create (and recreate) simulated environments “where the testing, training, teaching, and treatment of cognitive, emotional, and sensorimotor processes can take place under stimulus conditions that are not easily deliverable and controllable in the physical world” (Rizzo and Bouchard, 2019, p. 6). More specifically, numerous studies have demonstrated the efficacy of VR-based interventions to facilitate the expression and coping with emotions (e.g., Rizzo and Bouchard, 2019). Most of the virtual environments created and used as toll to treat potentially traumatic events are realistic in the sense that their aim is to virtually replicate, in a safe and controlled situation, a real life aversive situation (e.g., war environments, Peskin et al., 2019; sexual assault, Loranger and Bouchard, 2017). The virtual environment “EMMA World” was developed as a tool to facilitate the expression and coping with emotions in stress-related disorders (posttraumatic stress disorder, complicated grief, and adjustment disorders) (Baños et al., 2009, 2011). EMMA’s World objective is not to realistically reproduce the physical features of a threatening or traumatic event, but rather to employ symbols and personalized elements that can evoke emotional reactions and can facilitate the expression of patients’ emotional state in a symbolic way that will be used by the therapist. To achieve therapeutic objectives, different virtual landscapes and symbols are customized to be personally meaningful for the patient; those virtual elements are used to recreate, in a safe and controlled environment, emotions and situations that are difficult for the person to confront and process (Baños et al., 2011). The possibility to use personalized stimuli in EMMA World could be particularly relevant to support women in coping with their perinatal loss and the multitude of emotions that they can experience in this critical period of their life. It is expected that such a focus intervention would be more effective than services routinely provided and referred-to as treatment-as-usual (TAU).

The objective of the proposed paper is to describe the protocol of a randomized control trial aimed to assess a VR-based intervention for mothers who are experiencing perinatal loss. We hypothesize that: (a) when administered before TAU, the VR-based intervention will lead to a greater reduction in symptoms related to grief, postnatal depression and general psychopathology, compared to participants receiving TAU; (b) when administered after TAU, the participants will show a significant pre/post-VR-based intervention improvement in symptoms related to grief, postnatal depression and general psychopathology.

## Materials and Methods

### Inclusion/Exclusion Criteria

The intervention program will be offered to mothers who had recently experienced a perinatal death, including: having lost a child during pregnancy because of miscarriage (i.e., embryo or fetus death prior the 28th week of gestation); termination due to medical indications; stillbirth (i.e., fetus death after 28 completed weeks of gestation); or neonatal death (i.e., infant death within the first 7 days of life). Women who have experienced perinatal death while pregnant within no more than 1 year before the enrollment will be invited to participate to the study. Exclusion criteria include significant vision impairments despite wearing corrective glasses or lenses, presence of a diagnosed mental disorder, and being under psychological treatment. The target sample will be composed of 40 women. To evaluate the size of the involved samples, we used a Sample Size Calculation (Power Analysis) using the software GPower^∗^3. We based the power-analysis on a recent study assessing efficacy of an EMMA’s World-based intervention for the treatment of stress-related disorders. We estimated a minimum of 40 women to be included in the trial, in order to achieve a minimum power of 90%, considering a medium effect size of *f* = 0.25, and a significance level of 0.05.

### Measures and Data Analysis

#### Personal Information Sheet

Women will be asked about their age, nationality, relationship status, religion, education level, occupation, French proficiency, presence of psychiatric and/or physical problems (i.e., mobility and sight problems), being under psychological treatment and use of drugs. In addition, women will be asked to provide information about their perinatal loss: date of perinatal death, type of perinatal death (i.e., miscarriage, termination due to medical indications, voluntary termination of pregnancy, stillbirth, or neonatal death), gestation weeks, previous pregnancy, planned pregnancy, previous children.

#### Outcome Measures

Perinatal grief will be measured with the Perinatal Grief Scale (PGS; Potvin et al., 1989; Toedter et al., 2001; French validation: Paris et al., 2017). The PGS is composed by 33 statements divided into three subscales: Active Grief, which includes 11 items that refers to the normal emotional reactions to the loss (i.e., sorrow, missing the child, or crying); Difficulty Coping, which includes 11 items that refers to more complex emotional reactions (e.g., difficulty with normal life activities, lack of support, problems in marital relationships); and Despair, which refers to long-term effects of the loss (e.g., existential feelings of helplessness and hopelessness) and relevant coping strategies. The PGS uses a Likert type scale with five response options ranging from 1 (i.e., “strongly disagree”) to 5 (i.e., “strongly agree”). The total score varies between 33 and 165 points, with values above 91 points represent potential psychiatric morbidity. Complicated grief will be measured with the Inventory of Complicated Grief (IDC; Prigerson et al., 1995; French adaptation: Zech, 2006). This instrument consists of 19 items concerning the immediate bereavement-related thoughts and behaviors of the respondent, who is asked to report the frequency (from “0 = never” to “4 = always”) with which he/she currently underwent each of the emotional, cognitive, and behavioral states detailed in the instrument. Individuals with an ICG total scores > 25 reported to have significantly worse general, mental and physical health, social functioning, and bodily pain, as well as depression. Thus, the authors concluded that this score should be the criterion for distinguishing between uncomplicated and complicated grief reactions. Depressed mood will be assessed using the Beck Depression Inventory-II (BDI-II; Beck et al., 1996; Validated in French by Édition du centre de psychologie appliquée, 1996). The BDI-II is one of the most widely used instruments to assess depressive symptoms and their severity following the criteria of the Diagnostic and Statistical Manual of Mental Disorders (DSM-IV; American Psychiatric Association, 2000). The BDI-II consists in 21 symptoms assessed with multiple-choice responses rated on a scale value of 0–3. The maximum total score is 63, with the following standardized cutoffs: 0–13: associated to normal minimal range, 14–19: associated to mild depression, 20–28: associated to moderate depression, and 29–63: associated to severe depression. Postnatal depression will be assessed through the 10-item Edinburgh Postnatal Depression Scale (EDPE; Cox et al., 1987). The EPDS is the most commonly used screening tool to determine women’s clinically significant signs of depression during the perinatal period (e.g., McCabe-Beane et al., 2016). Since many symptoms of depression overlap with normal states in women during the perinatal period (e.g., sleep disturbance), EPDS items were planned to evaluate only the mood components of depressive symptoms. The 10 items are rated on a scale from 0 to 3, and are reflecting the mood during in the last 7 days. EPDS score of 10 and 13 are proposed as cut-off scores indicative of potential minor or major depression, respectively (Cox et al., 1987; see McCabe-Beane et al., 2016 for other cut-off scores). Anxiety will be assessed using the State and Trait Anxiety Inventory-form Y (STAI-Y; Spielberger, 1983; French validation: Gauthier and Bouchard, 1993). It consists of a self-report instrument conceived to evaluate trait (a stable personality trait) and state (a temporary and fluctuating condition) anxiety. The STAI-Y consists of two subscales (trait and state anxiety) with 20 items each rated on a 4-point scale. Higher scores indicate greater anxiety. Affect will be measured with the Positive and Negative Affect Scale (PANAS; Watson et al., 1988; French validation: Gaudreau et al., 2006). The PANAS is a widely used scale which consists of two subscales, one measuring positive affect and the other assessing negative affect. Each subscale is composed of 10 items, scored on a 5-point Likert scale ranging from 1 (i.e., “very slightly or not at all”) to 5 (i.e., “extremely”). Clinical global impression assessed by the therapist will be assessed at pretest and post-test using the Clinical Global Impression rated by the therapist (CGI; Guy, 1976). It consists in a 3-item observer-rated scale that measures illness severity (CGIS), global improvement or change (CGIC) and therapeutic response. The CGI is rated on a 7-point scale, with the CGIS characterized by a range of responses from 1 (i.e., “normal”) to 7 (i.e., “amongst the most severely ill patients”). CGI-C scores range from 1 (i.e., “very much improved”) to 7 (i.e., “very much worse”). Treatment response ratings should consider both treatment-related negative events and therapeutic efficacy, ranging from 0 (i.e., “marked improvement and no side-effects”) to 4 (i.e., “unchanged or worse and side-effects outweigh the therapeutic effects”). Each CGI’s component is rated separately, and this instrument does not provide a global score.

#### Satisfaction With the Intervention

Women’s satisfaction with the intervention will be rated at post-test using the Client Satisfaction Questionnaire (CSQ-8; Attkisson and Zwick, 1982). The CSQ-8 is a self-report measure designed to assess clients’ satisfaction with mental health services. The 8-items versions has eight question-items (quality of service, kind of service, met needs, recommend to a friend, amount of help, deal with problems, overall satisfaction, and come back), rated using a 4-point Likert scale, with a possible total scores can vary from 8 to 32. Higher scores indicate greater satisfaction with the service.

In order to test our hypothesis, we will perform repeated measures (Mixed Model) ANOVA with 3 Times (Pre, Post-test 1, Post-test 2) and 2 Conditions (VR-based intervention first, followed by TAU, i.e., VR+TAU; TAU first, followed by VR-based intervention, i.e., TAU+VR). For ethical reasons, the study is a randomized control crossover design, where all participants receive an intervention. The Pretest to Post-test 1 allows the comparison of VR-based treatment versus TAU, and the Post-test 1 to Post-test 2 allows to test the added value of the VR-Based intervention to TAU.

### Virtual Reality Setting

#### Devices

The following devices will be used: two computers, one projector, a wireless pad and a speaker system. One computer will have the graphical outputs from its graphic card connected to one projector (resolution of 1024_768 pixels and power of 2000 lumens). The projector will project the virtual environment on a screen of 4 × 1.5 m placed on a wall. Women will use the wireless pad in order to navigate and interact with the virtual environment. The second computer will permit to the therapist to adapt the virtual environment following the instruction of the participant (see Baños et al., 2009, 2011 for more detailed descriptions).

#### EMMA’s World Virtual Environment

**FIGURE 1:**
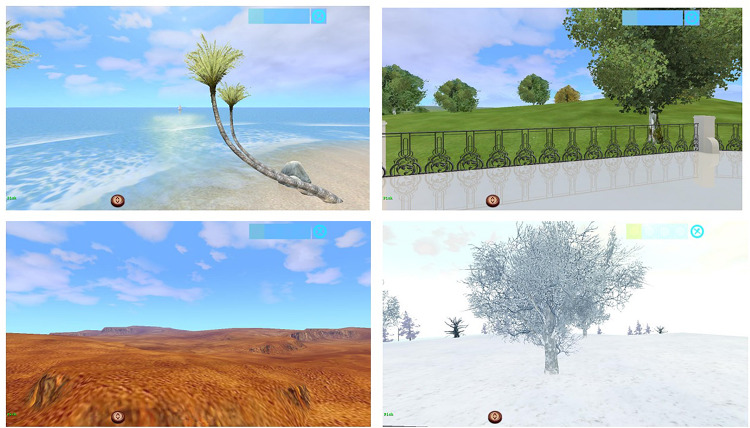
Examples of EMMA’s World landscapes.

**FIGURE 2:**
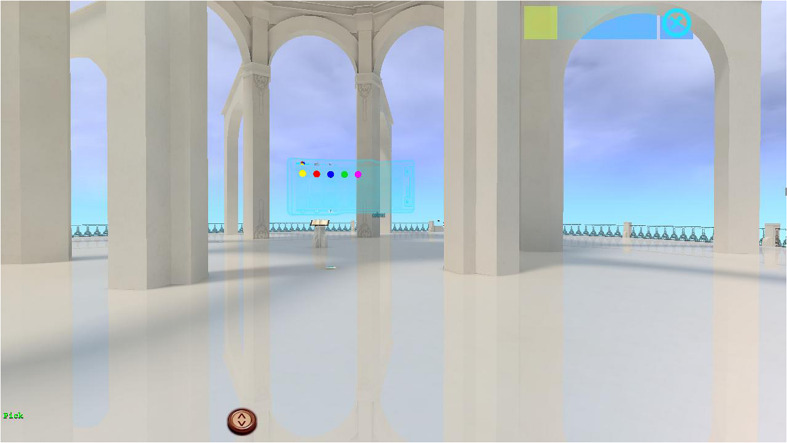
EMMA’s World temple.

Women will be able to choose between five different landscapes (i.e., beach/island, desert, meadow, dark forest, and snow-capped natural landscape) the one that better represent their emotional state related to the loss of their child (see Figure 1). These landscapes have been specifically designed to represent, in a metaphoric way, different emotions (e.g., joy, sadness, anxiety; Baños et al., 2009, 2011). Women will be able also to select some personalized virtual elements (e.g., three-dimensional objects, sound, images, and texts) which have been designed to help patients to express, confront and manage emotions and difficult life experiences (for a more detailed description please refer to Baños et al., 2009, 2011). At the center of each landscape there is a virtual temple (Figure 2), where women can have access to the “Book of Life,” a virtual book in which women will be able to reflect and write their feelings and thoughts related to their loss. Patients are not only able to write, but also to insert personal pictures, three-dimensional symbols, videos, and sounds, in their Book of Life. This book has different chapters that can define the progresses of the intervention and/or can represent the different chapters of the women’s life. Finally, the time of the day in the virtual environment can also be customized. The therapist can control all of these elements from a single interface.

#### VR Intervention

The proposed intervention will consist in three weekly sessions of 2 h focused, respectively, on: (1) gathering information about the loss and psychoeducation about perinatal grief, and introduction to EMMA World; (2) through the use of EMMA World, women will be assisted in the elaboration and acceptation of loss; (3) recreate, using the features of EMMA World (i.e., a series of different virtual landscapes and symbolic elements personalized to each participant as personal pictures, sounds or videos) a new and positive metaphor representing the woman’s future life.

### Procedure

**FIGURE 3:**
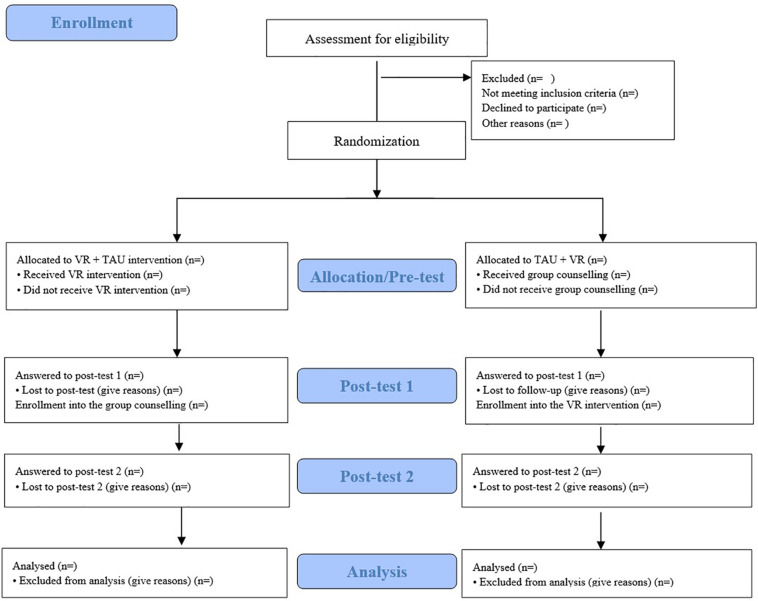
CONSORT flowchart for the proposed clinical trial: adapted from Consort 2010.

Women will be recruited at Université du Québec en Outaouais (UQO) as well as at the Centre d’études et de recherché en intervention familiale (CERIF) and at the Association Parents Orphelins^1^. In a first screening session, mothers will be informed about the study, asked to agree and sign a consent form and will be also asked to answer to a battery of questionnaires (i.e., “Assessment for eligibility, Figure 3). Information about the history of participant’s pregnancy and perinatal loss will also be collected during this first in-person meeting.

Women will be randomly assigned to the VR+TAU or to the TAU + VR condition. Randomization will be performed using a true random number service^2^, and will be not stratified based on participant’s characteristics. The TAU condition is a homogenous standard program proposed by the CERIF at UQO based on group counseling offered to women and men who experienced a perinatal loss. The TAU condition as well as the VR-based intervention will last 3 weeks, after which women will complete a post-assessment (see Figure 3). The VR-based intervention will be conducted at the Cyberpsychology Laboratory of UQO; the TAU group counseling will take place at the CERIF of UQO.

## Discussion

The aim of the proposed study is to assess a VR-based intervention for mothers after pregnancy loss in a randomized controlled trial. In order to test our hypotheses, we will compare the innovative VR-based intervention presented in this paper, with a TAU condition. Specifically, we hypothesize that the VR-based intervention group will show significantly reduced symptoms related to grief, postnatal depression and general psychopathology after treatment relative to a TAU group.

Despite the potential emotional impact and significant prevalence of loss during pregnancy, in Québec there are no specific protocols for providing psychological and emotional support to those mothers (de Montigny et al., 2017b). VR has proved to be a valid intervention tool in clinical psychology, and in the last years VR technologies have become more affordable to be used in clinical practice (Rizzo and Bouchard, 2019). The present study proposes to answer to the need for interventions designed for women who experienced a complex experience as a perinatal loss and make good use of the therapeutic opportunities offered by VR. If effective, the proposed VR-based intervention could be implemented in clinical care practice, giving the opportunity to women who experienced a perinatal death to access to well-established effective psychological intervention. In order to maintain a homogeneous sample for this first study, the intervention will be provided only to woman who have experience perinatal death. This excludes men and other people who might have been closely involved in the pregnancy (e.g., a female spouse). Future studies should also take in consideration men’s experiences and non-traditional parenthood. Indeed, the loss of a child can cause a multitude of complex emotions and psychological distress in mothers as well as in fathers. However, fathers’ experiences of perinatal loss have been less investigated comparing to the mother’s ones (de Montigny et al., 2017a). Since in this study we have focused on women’s experience of perinatal loss, future studies should also investigate to applying this VR-based intervention to men who are dealing with the loss of their baby during the perinatal period.

## Ethics Statement

The proposed study was approved by the Ethical Committee of UQO, and will be conducted in accordance with the CONSORT 2010 Statement and the Helsinki Declaration of 1975, as revised in 2018.

## Author Contributions

GC, SB, and FM conceived and designed the protocol. RB participated in the design and developed the virtual environment. GC wrote the first draft. SB, FM, RB, and CV provided the required revisions. SB, FM, RB, and CV were GC supervisors. All authors revised and approved the final version of the manuscript.

## Conflict of Interest

SB was consultant to and owns equity in Cliniques et Développement In Virtuo, a spin-off from the university that uses virtual reality as part of its clinical services and distributes virtual environments. The terms of these arrangements have been reviewed and approved by the Université du Québec en Outaouais in accordance with its conflict of interest policies. The remaining authors declare that the research was conducted in the absence of any commercial or financial relationships that could be construed as a potential conflict of interest.

## Footnotes

1 https://parentsorphelins.org/

2 http://www.random.org
